# Gene Signatures and Cancer-Immune Phenotypes Based on m^6^A Regulators in Breast Cancer

**DOI:** 10.3389/fonc.2021.756412

**Published:** 2021-11-04

**Authors:** Guanghui Zhao, Junhua An, Qian Pu, Wenwen Geng, Haiyun Song, Qianqian Zhao, Haidong Gao

**Affiliations:** ^1^ Medical Laboratory Center, Qilu Hospital (Qingdao), Cheeloo College of Medicine, Shandong University, Qingdao, China; ^2^ Department of Breast Surgery, Qilu Hospital (Qingdao), Cheeloo College of Medicine, Shandong University, Qingdao, China; ^3^ Department of Pathology, Qilu Hospital (Qingdao), Cheeloo College of Medicine, Shandong University, Qingdao, China; ^4^ Department of Breast Surgery, Qilu Hospital, Cheeloo College of Medicine, Shandong University, Jinan, China

**Keywords:** breast cancer, m^6^A regulators, tumor immune microenvironment, prognostic biomarkers, immune phenotype

## Abstract

The N^6^-methyladenosine (m^6^A) has been considered as a new layer of epitranscriptomic regulation on mRNA processing, stability, and translation. However, potential roles of m^6^A RNA methylation modification in tumor immune microenvironment (TIME) of breast cancer are yet fully understood. In this study, we comprehensively evaluated the genetic variations and transcript expressions of 15 m^6^A regulators in 1,079 breast cancer samples from the Cancer Genome Atlas (TCGA) database. We validated major regulators had significantly differential mRNA and protein expression in tumor tissue compared to normal tissues from 39 pairs of clinical breast cancer samples with different molecular subtypes, and especially high expression of m^6^A readers YTHDF1 and YTHDF3 predicted poor survival. Two clusters of breast cancer patients identified by the 15 m^6^A regulators’ pattern showed distinct overall survival, immune activation status, and immune cell infiltration, and clinical samples confirmed the diversity of lymphocytic infiltration. The profiles of these two clusters accorded with that of two classical cancer-immune phenotypes, immune-excluded and immune-inflamed phenotypes, it suggested that m^6^A regulators-based patterns might serve as crucial mediators of TIME in breast cancer. Moreover, the m^6^A phenotype-related gene signatures could also be survival predictor in breast cancer. Therefore, comprehensive evaluation of tumor m^6^A modification pattern will contribute to enhance our understanding of the characterization of immune cell infiltration in the tumor microenvironment and promote the responsiveness of breast cancer to immunotherapy.

## Introduction

Breast cancer, the most frequent malignancy in women, will affect as many as one in eight women in high-income countries by age 85 years (1). In 2020, female breast cancer has surpassed lung cancer as the most commonly diagnosed cancer. About 2.3 million women were newly diagnosed with breast cancer, and 684,996 women with breast cancer died (2). In high-income countries, breast cancer is often diagnosed at an early stage and the prognosis is usually good. However, in low- or middle-income countries, breast cancer is often diagnosed at an advanced stage with poorer survival (3). Breast cancer is a heterogeneous disease on the molecular level due to the activation of different molecular features or gene alterations (4). Diverse immune microenvironment also contributes to the heterogeneity, and influences the progression and therapeutic response of breast cancer (5). Breast cancer with infiltrating immune cells is known to have better survival and higher response to neoadjuvant chemotherapy or immunotherapy (6); however, less is known about the underlying mechanisms and associated immune phenotypes. Therefore, it is necessary to comprehensively profile the heterogeneity and complexity of tumor immune microenvironment (TIME) landscape and identify different tumor immune phenotypes in breast cancer.

N^6^-methyladenosine (m^6^A), methylated adenosine at the N^6^ position, is the most prevalent internal modification in mRNA of eukaryotic species (7). Similar to DNA and protein, RNA can be methylated and demethylated by different methylation regulators, including methyltransferases (also known as “writers”) and demethylases (also known as ‘‘erasers’’). Modified RNAs can be further recognized by “readers” proteins (8). The deposition of m^6^A modifications in mRNAs is executed by a multicomponent methyltransferase complex, including METTL3, METTL14 and WTAP, and so on (9, 10). The removal of m^6^A could be realized by FTO and ALKBH5 (11, 12), and readers, like YTH domain–containing proteins, mediate the regulatory functions of m^6^A on modified RNAs (13, 14). As a reversible epigenetic modification, these m^6^A regulators affect the fate of the modified RNA molecules and play important roles in the tumorigenesis and progression of multiple cancers, including breast cancer (8). Niu et al. found that FTO promoted tumor progression by mediating m^6^A demethylation in the 3’UTR of BNIP3 mRNA in human breast cancer (15). Cai et al. identified that METTL3 increased HBXIP expression by forming a positive feedback loop of HBXIP/let-7g/METTL3/HBXIP, eventually leading to accelerated cell proliferation in breast cancer (16). Another member of methyltransferases, METTL14, could be recruited by long non-coding RNA (lncRNA) LNC942 and promoted breast cancer initiation and progression by stabilizing the expression of downstream targets of LNC942 including CXCR4 and CYP1B1 through posttranscriptional m^6^A methylation modification (17). However, the potential biological functions of other m^6^A regulators, especially most of m^6^A readers, have not been comprehensively clarified in breast cancer.

In recent years, studies have shown that RNA m^6^A modification is involved in host antitumor immune responses. Ythdf1-deficient mice showed an elevated antigen-specific CD8^+^ T cell antitumor response, and loss of YTHDF1 in classical dendritic cells enhanced the cross-presentation of tumor antigens and the cross-priming of CD8^+^ T cells *in vivo*. YTHDF1 recognized transcripts encoding lysosomal proteases to increase the translation in dendritic cells *via* an m^6^A dependent manner. Moreover, the therapeutic efficacy of PD-L1 checkpoint blockade was enhanced in Ythdf1-deficient mice, implicating YTHDF1 as a potential therapeutic target in anticancer immunotherapy (18). Wang et al. recently found that Mettl3 and Mettl14 enhanced response to anti-PD-1 treatment in colorectal cancer and melanoma. Mettl3- or Mettl14-deficient tumors increased cytotoxic tumor-infiltrating CD8+ T cells and elevated secretion of IFN-c, Cxcl9, and Cxcl10 in tumor microenvironment *in vivo* (19). Su et al. identified two potent FTO inhibitors and demonstrated that targeting the FTO/m^6^A axis could significantly suppress cancer stem cell self-renewal and immune evasion by suppressing expression of immune checkpoint genes, especially LILRB4. Targeting FTO by potent inhibitors held therapeutic promise against various types of cancers, including breast cancer (20). Yang et al. demonstrated that FTO inhibition suppresses melanoma tumorigenicity and the expression of melanoma cell-intrinsic genes PD-1 (PDCD1), CXCR4, and SOX10 through an m^6^A dependent, YTHDF2-mediated mRNA decay. Knockdown of FTO sensitized melanoma cells to interferon gamma and sensitized melanoma to anti-PD-1 treatment in mice, depending on adaptive immunity (21). However, there is still a lack of researches on the mechanism of m^6^A modification involved in antitumor immune response in breast cancer.

In this study, we aimed to comprehensively characterize the genetic variations of multiple m^6^A regulators and the correlation of m^6^A regulators’ expression and immune infiltration in breast cancer. Therefore, we integrated the genomic and transcriptomic information of 15 m^6^A regulators from more than 1,000 breast cancer samples to evaluate m^6^A regulators’ mutations, expression pattern, and the relationships between clustering subtypes, clinicopathological characteristics, and immune microenvironment based on TCGA database. Most of m^6^A regulators revealed differential mRNA and protein expression between tumor and normal tissue in our clinical cohort of breast cancer patients. High expressions of YTHDF1 and YTHDF3 were related to poor survival of patients with breast cancer. Two distinct clustering subsets uncovered by 15 m^6^A regulators had different immune activation status and might be associated with two cancer-immune phenotypes. Our study elucidated the important role of m^6^A modification in immune microenvironment of breast cancer, and provided new insights into the regulatory mechanisms of m^6^A regulators involved in breast cancer immunotherapy.

## Materials and Methods

### Breast Cancer Dataset Source

The genomic, transcriptomic, and clinical data of this study were downloaded from 1,090 breast cancer in TCGA database (https://portal.gdc.cancer.gov/). For genomic data, 986 and 1,067 samples were used for somatic mutation and copy number variation (CNV) analysis of m^6^A regulators, respectively. For gene expression data, 1,079 breast samples with corresponding clinicopathological information, including gender, TNM stage, pathologic stage, and survival status, were downloaded for consensus clustering analysis. Among them, 112 breast cancer and paired adjacent normal samples were adopted to analyze differential expression of m^6^A regulators in groups. For clinical correlation analysis, only those samples with complete clinicopathological data were extracted in different types of grouping.

The Gene Expression Profiling Interactive Analysis (GEPIA) database was adopted to validate the differential expression of m^6^A regulators (http://gepia.cancer-pku.cn/index.html). The GEPIA was an interactive web server for analyzing the RNA sequencing expression data, and collected more samples from the TCGA and the GTEx projects.

### m^6^A Methylation Regulators Analysis

According to previously published literature, the most widely studied 15 m^6^A regulators, including six writer complexes (METTL3, METTL14, METTL16, WTAP, VIRMA, RBM15), two erasers (FTO, ALKBH5), and seven readers (YTHDC1/2, YTHDF1/2/3, HNRNPA2B1, EIF3A), were chosen in this study (8, 22). Genetic variation and differential expression analysis of these 15 m^6^A regulators were performed based on the TCGA data.

### Correlation of CNV Pattern and Gene Expression

To investigate the effects of CNV on gene expression, the CNV patterns of 15 m^6^A regulators were divided into deep deletion, shallow deletion, diploid, copy number gain, and amplification in 1,059 breast cancer samples. The relative expression levels of 15 m^6^A regulators were used for analyzing the relationship between mRNA expression and CNV.

### Clinical Breast Cancer Samples

Thirty-nine pairs of frozen breast tumor and matched adjacent samples were obtained from Qilu Hospital with all patients’ informed consent. This study was also approved by the Ethics Committee of Qilu Hospital of Shandong University (Qingdao). The clinical data of 39 patients was shown in **Supplementary Table S6**. Thirty-three pairs of breast tissues were used for qRT-PCR analysis of m^6^A regulators. Six paired samples of tumor and adjacent tissues were used for protein expression analysis of five major regulators by (IHC), and all 39 paired samples were used for expression analysis of YTHDF1 and YTHDF3 by IHC. Seven paired samples used in qRT-PCR validation were adopted for western blot analysis.

### RNA Extraction, Reverse Transcription, and qRT-PCR

Total RNA was extracted from the tumor and matched adjacent tissue samples using TRIzol LS reagent (Thermo Fisher Scientific, USA), and the cDNA was synthesized by Reverse Transcriptase M-MLV (Takara, Japan) following the manufacturer’s instructions. In addition, qRT-PCR was performed with SYBR^®^ Premix Ex Taq™ II (Takara, Japan). GAPDH was used as endogenous control for m^6^A regulators’ qRT-PCR. All primers of several regulators were listed in **Supplementary Table S9**. The relative regulators’ expression was compared using 2^−ΔCt^ between tumor and adjacent samples, with ΔCt = Ct_regulator_ – Ct_GAPDH_.

### Western Blot

For western blot, 100 mg of fresh tissues were isolated, homogenized, and added into RIPA Lysis Buffer (Thermo Scientific, USA). After ultrasonic treatment, the proteins of the lysated samples were prepared. A total of 30 μg of protein per sample was quantified and separated by SDS-PAGE, then electrically transferred to a polyvinylidene fluoride (PVDF) membrane (Millipore, USA). The primary antibodies of METTL3 (Cat. No. 382974, Zen bioscience, China), METTL14 (Cat. No. 508530, Zen bioscience, China), FTO (Cat. No.R24362, Zen bioscience, China), YTHDF1 (Cat. No. A18126, ABclonal, China), YTHDF3 (Cat. No. A8395, ABclonal, China), and β-actin (Cat. No. ab8227, Abcam, UK) were diluted in corresponding proportion (**Supplementary Table S10**) and incubated with the PVDF membrane, then incubated with the secondary antibodies labeled by HRP (Cat. No. ab6721, Abcam, UK). ECL chemiluminescence method was used for testing.

### IHC

For IHC, the slides were deparaffinized, rehydrated at room temperature after roasting, then placed in a pressure cooker for antigen retrieval. After natural cooling, antigen sealing was performed. The primary antibodies of METTL3and METTL14 were diluted at 1:100, FTO was diluted at 1:20, and YTHDF1 and YTHDF3 were diluted at 1:200, incubated at room temperature, then the secondary antibodies were added for incubation. DAB was added for color development, and the slides were finally counterstained with hematoxylin, dehydrated, and mounted with Permount (Thermo Fisher Scientific, USA). Protein expression levels were analyzed by an automatic section scanning system (Roche, USA) and matching analysis software.

### Prognosis Analysis

The UALCAN online tool (http://ualcan.path.uab.edu) was a comprehensive, user-friendly, and interactive web resource for analyzing cancer OMICS data. The UALCAN tool was adopted to analyze the relationship of gene expression and breast cancer patient survival information based on gene expression levels of m^6^A regulators based on the TCGA database.

The Human Protein Atlas (HPA, https://www.proteinatlas.org/) was used to validate the correlation between m^6^A regulators’ expression levels and breast cancer patients’ survival. The prognosis of each group of patients was examined by Kaplan-Meier survival estimators, and the survival outcomes of the two groups were compared by log-rank tests.

The Kaplan-Meier Plotter analysis tool (http://kmplot.com/analysis/) was used to further access the effect of m^6^A regulators’ expression levels on the prognosis of patients with breast cancer. The Kaplan-Meier Plotter collected more sample source from multiple databases including GEO, EGA, and TCGA.

### Consensus Clustering Analysis

To functionally identify distinct m^6^A modification patterns based on the expression of 15 m^6^A regulators in breast cancer, we employed the “ConsensusClusterPlus” package (1,000 iterations and resample rate of 80%, http://www.bioconductor.org/) to classify the patients with breast cancer into different subtypes. The number of clusters and their stability were determined by the consensus clustering algorithm.

### Estimation of TIME Cell Infiltration

The immunoscore for each patient was calculated with the ESTIMATE algorithm through the R “estimate package” (23). The fraction of 22 immune cell types for each sample was yielded by estimating relative gene subsets of RNA transcripts in different cell types (CIBERSORT; https://cibersort.stanford.edu/). The algorithm of 1,000 permutations was adopted. Only samples with a CIBERSORT p < 0.05 were included to perform the subsequent analysis of comparing differential immune infiltration levels between the subgroups grouped by clustering subtypes.

### Gene Set Enrichment Analysis

The gene set enrichment analysis (GSEA) was provided by the JAVA program with Molecular Signatures Database (MSigDB) v7.1 and download from the website of Broad Institute (https://www.gsea-msigdb.org/gsea/index.jsp). Then, differentially enriched hallmark gene sets between the two groups were defined by an effect size of normalized enrichment score (NES) differences being greater than 1.5 and nominal p-value < 0.05.

### Identification of Differential Genes Between Distinct m^6^A Modification Phenotypes

The previous consensus clustering classified breast cancer patients into two distinct m^6^A modification patterns, and we next determined m^6^A modification-related differentially expressed genes (DEGs) among these two m^6^A phenotypes. The R package “limma” was used to evaluate DEGs in breast cancer samples between different modification clusters. The strict filtering criteria of DEGs were set as an adjusted p-value less than 0.001.

### Statistical Analysis

The association between m^6^A regulatory genes’ CNV and clinicopathological characteristics were analyzed with chi-square test or Student’s t test. The expression levels of the m^6^A RNA methylation regulators were compared with the Mann-Whitney U test in breast cancer tissues *versus* paired normal tissues. Student’s t test was used to perform difference comparison of two groups. Survival curves were generated using the Kaplan-Meier method with calculated hazard ratio with the 95% CI, and the difference between groups was compared with the log rank test. Univariate analysis was conducted using Cox regression model to determine the independent prognostic value of 15 m^6^A regulators in breast cancer, and multivariate analysis was performed to test the independent prognostic value of the clusters and other clinical variables. Spearman correlation analysis was performed among 15 m^6^A regulators and 22 infiltration cell types. All statistical results with a p-value < 0.05 were considered to be significant. All data processing was done in R 3.6.1 software.

## Results

### Landscape of Genetic Variations of m^6^A Regulators in Breast Cancer

**Figure 1:**
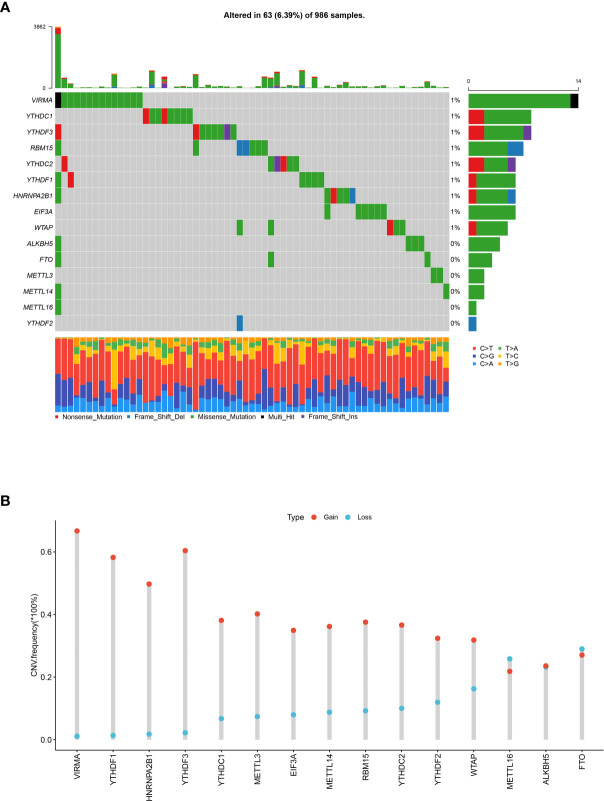
Landscape of somatic mutations and CNVs of 15 m^6^A regulators in breast cancer. **(A)** The waterfall plot of tumor somatic mutations of 15 m^6^A regulators in 986 breast cancer patients. Each column represented a sample or patient, and mutation rates in the tumor samples were shown in the top barplot. The number on the right indicated the mutation frequency of each regulator, and the right barplot showed the proportion of each variant type. The bottom barplot represented the proportion of each base mutation in each sample. **(B)** The CNVs frequency of 15 m^6^A regulators in 1,067 breast cancer samples. The height of the column represented the variation frequency. The blue dot was deletion frequency; the red dot was amplification frequency.

To evaluate the biological functions of m^6^A regulators in breast cancer, a total of 15 m^6^A regulators including six writer complexes (METTL3, METTL14, METTL16, WTAP, VIRMA, RBM15), two erasers (FTO, ALKBH5), and seven readers (YTHDC1/2, YTHDF1/2/3, HNRNPA2B1, EIF3A) were investigated based on available TCGA dataset. We first assessed the frequency of somatic mutations and CNVs of 15 m^6^A regulators in breast cancer. Among 986 samples, only 63 (6.39%) samples had mutation events of m^6^A regulators. Several members of m^6^A writer complexes and readers exhibited 1% mutation frequency, while main methyltransferases (METTL3, METTL14) and demethylases (FTO, ALKHB5) did not mutate in breast cancer samples (**Figure 1A**). However, it was found that 15 regulators had prevalent CNV alteration events, and most showed higher frequency of CNV amplification in 1,067 breast cancer samples. The writer gene VIRMA (67.85%, 724/1,067) harbored the most CNV events among the 15 regulators, followed by reader gene YTHDF3 (62.70%, 669/1,067) and YTHDF1 (59.70%, 637/1,067, **Figure 1B**). The location of CNV alterations of m^6^A regulators on chromosomes was shown in **Supplementary Figure S1A**. Then, we intended to know whether the global CNV alterations of 15 regulators were associated with the clinicopathological characteristics of breast cancer patients. The results revealed that global CNV alteration events of m^6^A regulators had no correlations with patients’ age, gender, pathological stage, TNM stage (**Supplementary Table S1**), and overall survival (p = 0.89, **Supplementary Figure S1B**). However, single regulator’s CNV analysis showed that the CNV alterations were associated with pathological stage and T stage, including VIRMA, YTHDF1, and YTHDF3 (**Supplementary Tables S2–S5**). We unexpectedly found the most significant correlation in METTL14’s CNV and patients’ T stage (p = 9.66E-05). Therefore, some m^6^A regulators’ CNV events might be potential biomarkers of patient’s stage in breast cancer.

### Differential mRNA Expression Pattern of m^6^A Regulators in Breast Cancer

**Figure 2:**
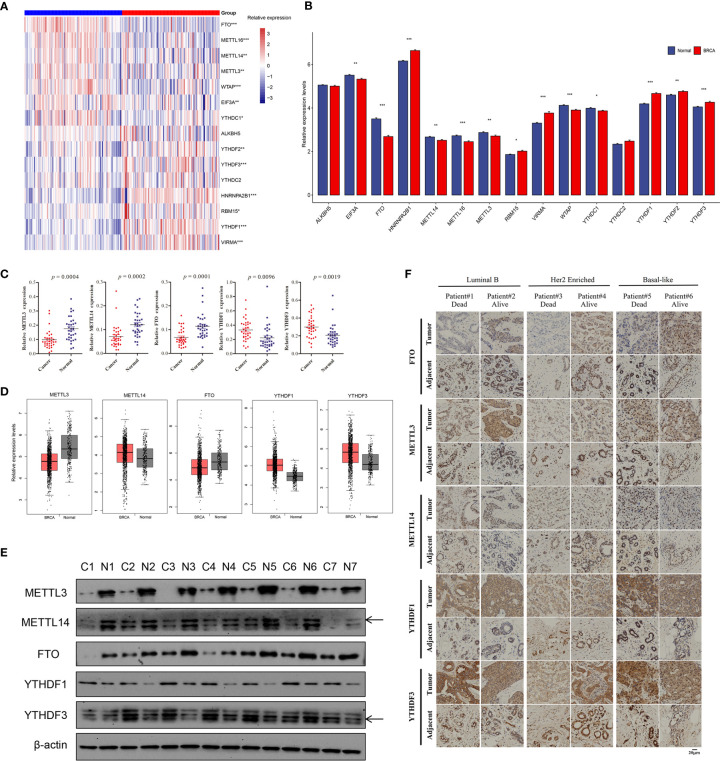
Relative mRNA and protein expression levels of m^6^A regulators in breast cancer. **(A)** The heatmap of 15 m^6^A regulators’ expression levels in 112 paired breast cancer samples; red, breast cancer; blue, adjacent normal samples. **(B)** The barplot of relative expression levels of m^6^A regulators in paired samples; BRCA, breast cancer. **(C)** The qPCR validation results of five significantly differential m^6^A regulators in 33 paired clinical samples of breast cancer patients. **(D)** The relative expression levels of five m^6^A regulators in tumor and normal samples based on GEPIA analysis, p < 0.01. **(E)** The western blot results of five m^6^A regulators in seven paired tumor and adjacent samples, and β-actin was used as endogenous control. **(F)** The IHC of five m^6^A regulators in six patients with different molecular subtypes and survival state. The asterisks represented the statistical p-value, *p < 0.05, **p < 0.01, ***p < 0.001.

To assess whether the above genetic variations affected gene expression levels of m^6^A regulators in breast cancer patients, we first compared the mRNA levels between 112 paired breast cancer and adjacent normal samples based on TCGA data, and found that most m^6^A regulatory genes were significantly different in breast cancer and normal samples (p < 0.05), except for YTHDC2 (p = 0.083) and ALKBH5 (p = 0.092, **Figures 2A, B**). Among them, the expression of RBM15, VIRMA, HNRNPA2B1, and YTHDF1/2/3 were significantly upregulated, but METTL3, METTL14, METTL16, WTAP, FTO, YTHDC1, and EIF3A had lower expression in breast cancer compared to normal samples (**Figures 2A, B**). FTO had the most significant difference with downregulation in paired tumor samples (p = 7.10E-19). We chose some regulators for RT-qPCR validation, and the qPCR results revealed that METTL3, METTL14, and FTO had consistently low expression in 33 paired samples with TCGA data, while YTHDF1 and YTHDF3 showed increased expression in tumor samples compared to adjacent tissues (**Figure 2C**). We adopted the GEPIA analysis tool, which included more normal samples from the GTEx projects, to analyze the expression difference of these five regulators, and it showed that METTL3 and FTO were significantly downregulated in breast tumor samples, YTHDF1 and YTHDF3 were significantly upregulated in tumor tissues (**Figure 2D**). Therefore, these public data and our experimental results validated differential mRNA expression of several regulators in breast cancer. Furthermore, western blot revealed that the protein levels of METTL3, METTL14, and FTO were significantly lower in seven breast tumor samples randomly selected from RT-qPCR samples, while YTHDF1 and YTHDF3 had higher protein expression compared to adjacent tissues (**Figure 2E**). Six paired samples from two luminal B, two Her2 enriched, and two triple-negative breast cancer (TNBC) patients were used for IHC of METTL3, METTL14, FTO, YTHDF1, and YTHDF3. These breast cancer patients with distinct molecular subtypes all represented lower expression of FTO, METTL3, and METTL14, and higher expression of YTHDF1 and YTHDF3 in tumor compared to the corresponding adjacent samples (**Supplementary Table S6** and **Figure 2F**). Furthermore, the deceased patients had relatively reduced expression of FTO, METTL3, and METTL14 in tumor tissues, and increased levels of YTHDF1 and YTHDF3 than the alive patients who were diagnosed with breast cancer at the same period (**Figure 2F**). All 39 paired samples were used for expression validation of YTHDF1 and YTHDF3, and the results revealed that YTHDF1 and YTHDF3 had higher expression in 32 and 30 tumor samples compared to the corresponding adjacent tissue, respectively (**Supplementary Files 1** and **2**). These results suggested consistent mRNA and protein expression difference of major m^6^A regulators in breast cancer.

**Figure 3:**
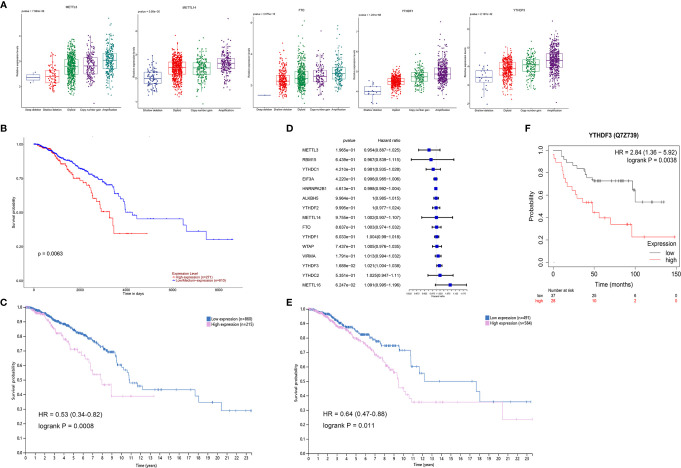
The survival correlation of YTHDF1 and YTHDF3 in breast cancer based on the online database. **(A)** The correlations between different CNV patterns and mRNA expression levels of five m^6^A regulators; other regulators were represented in **Supplementary Figure S2**. **(B)** Overall survival analysis for high and low/medium expression of YTHDF1 using Kaplan-Meier curves by UALCAN analysis, p = 0.0063. **(C)** The correlation of YTHDF1 expression and patients’ survival in breast cancer based on the HPA, p = 0.0008. **(D)** The prognostic analyses for 15 m^6^A regulators in breast cancer based on TCGA database using a univariate Cox regression model. Hazard ratio >1 represented risk factors for survival. **(E)** The correlation of YTHDF3 expression and patients’ survival in breast cancer based on the HPA, p = 0.011. **(F)** The correlation of YTHDF3 protein expression and patients’ survival in breast cancer by Kaplan-Meier Plotter tool, p = 0.0038.

Considering the relationship between genetic variations and gene expression, the effects of CNV alterations in m^6^A regulators on the mRNA expression were analyzed (detailed information in supplementary materials). The results showed that the mRNA levels of all genes were significantly associated with diverse CNV patterns in 1,059 breast cancer samples; CNV gain or amplification was related to higher expression; however, shallow or deep deletion resulted in lower mRNA levels (**Figure 3A** and **Supplementary Figure S2**). The UALCAN online tool was used to analyze the prognostic value of 15 m^6^A regulators based on medium expression levels from 1,081 breast cancer patients, and just identified that high mRNA expression of YTHDF1 was associated with poor survival (p = 0.0063, **Figure 3B**). The HPA analysis also validated that YTHDF1 was prognostic, and its high mRNA expression was unfavorable in 1,075 breast cancer samples (p = 0.0008, **Figure 3C**). A univariate Cox regression model revealed the prognostic values of 15 m^6^A regulators in patients with breast cancer, and only YTHDF3 had prognostic significance (**Figure 3D**). Interestingly, the HPA data showed that patients with high YTHDF3 expression also had poorer survival probability (p = 0.011, **Figure 3E**). Moreover, the Kaplan-Meier Plotter online database revealed high expression of YTHDF3 protein predicted poor prognosis (p = 0.0038, **Figure 3F**). In addition, we evaluated the prognostic roles of YTHDF1 and YTHDF3 in different molecular subtypes of breast cancer from HPA data and found that high expression of YTHDF1 was significantly related with poor prognosis in Her2 enriched and luminal B subtypes, while high expression of YTHDF3 was significantly correlated with poor prognosis in Her2 enriched, luminal A, and luminal B subtypes (**Supplementary Figure S3**). Therefore, these results suggested that m^6^A reader YTHDF1 and YTHDF3 might be potential survival biomarkers of breast cancer.

### Significant Correlation of Consensus Clustering for m^6^A Regulators With the Survival of Breast Cancer Patients

**Figure 4:**
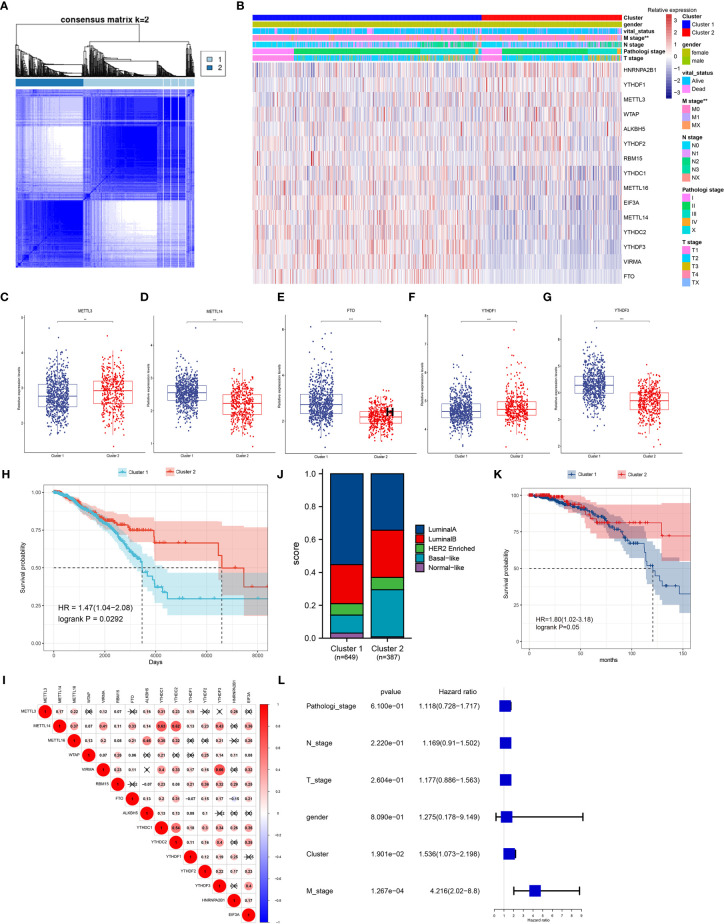
Patterns of 15 m^6^A regulators and clinicopathological features in TCGA cohort. **(A)** Consensus clustering matrix for k = 2. **(B)** Unsupervised clustering of 15 m^6^A regulators and clinicopathological features of 1,079 breast cancer patients from TCGA data. The gender, survival status, TNM stage and pathological stage were used as patient annotations. **(C–G)** The relative expression levels of METTL3 **(C)**, METTL14 **(D)**, FTO **(E)**, YTHDF1 **(F)** and YTHDF3 **(G)** between cluster 1 and cluster 2, other regulators were represented in **Supplementary Figure S4**. *p < 0.05, **p < 0.01, ***p < 0.001. **(H)** Kaplan-Meier curves of overall survival for patients with breast cancer in two clusters. **(I)** Spearman correlation analysis of the 15 m^6^A methylation regulators, positive correlation was marked with red. **(J)** The clinical subtypes of breast cancer patients in cluster 1 and cluster 2 based on the PAM50. **(K)** Kaplan-Meier curves of overall survival for patients with luminal A subtype in two clusters. **(L)** The multivariate Cox regression model analysis of different clinical variables.

Considering the correlation of gene expression and the clinicopathological characteristics of breast cancer patients, consensus clustering of the 15 m^6^A regulators was performed in breast cancer patients. The k = 2 was identified with optimal clustering stability from k = 2 to 9 based on the similarity displayed by the expression levels of m^6^A regulators and the proportion of ambiguous clustering measure (**Figure 4A**). Total 1,079 breast cancer patients were clustered into two subtypes, named, cluster 1 (n = 669) and cluster 2 (n = 410), based on the mRNA levels of the m^6^A regulators (**Figure 4B**). Most m^6^A regulators were differentially expressed in two clusters, and high expression of METTL14, VIRMA, METTL16, FTO, EIF3A, YTHDC1/2, and YTHDF3 were shown in cluster 1 (**Figures 4C–G** and **Supplementary Figure S4**). The overall survival of cluster 2 was longer than those of cluster 1 (p = 0.029, **Figure 4H**). Therefore, consensus clustering of these m^6^A regulators could serve as a potential prognostic factor for breast cancer. However, other clinicopathological features between the two subtypes did not have significant differences except for M (metastasis) stage of patients (p = 0.0065, **Figure 4B** and **Supplementary Table S7**). The finding suggested the clustering subsets defined by 15 m^6^A regulators’ expression might be due to the heterogeneity of breast cancer patients. To further explore the interaction among these regulators, we analyzed the correlations of 15 m^6^A regulators (**Figure 4I**). It could be found that the expression levels of METTL14, VIRMA, RBM15, YTHDC1/2, YTHDF1/2/3 were positively correlated with each other (p < 0.05). The m^6^A reader YTHDF3 had the most significant correlation with m^6^A writer complex VIRMA, followed by YTHDC1/2 with METTL14 (p < 0.05). These results suggested possible functional links between m^6^A readers and writers in breast cancer.

Interestingly, the TCGA samples could be classified into five recognized subtypes according to the PAM50 classifier (24), including luminal A, luminal B, basal-like, Her2 enriched, and normal-like (**Figure 4J**). Each clinical subtype was reclassified using the two clusters defined by 15 m^6^A regulators, and the results showed that there was a significant difference in overall survival between the two groups in the luminal A subtype (p = 0.05, **Figure 4K**). The difference began at about 75 months and became significant at 150 months, up to about 220 months. A multivariate Cox regression model revealed that the clusters had relatively higher hazard ratio with most of the clinical variables (p = 0.019, **Figure 4L**).

### Consensus Clustering for m^6^A Regulators Associated With Distinct Cancer-Immune Phenotypes

**Figure 5:**
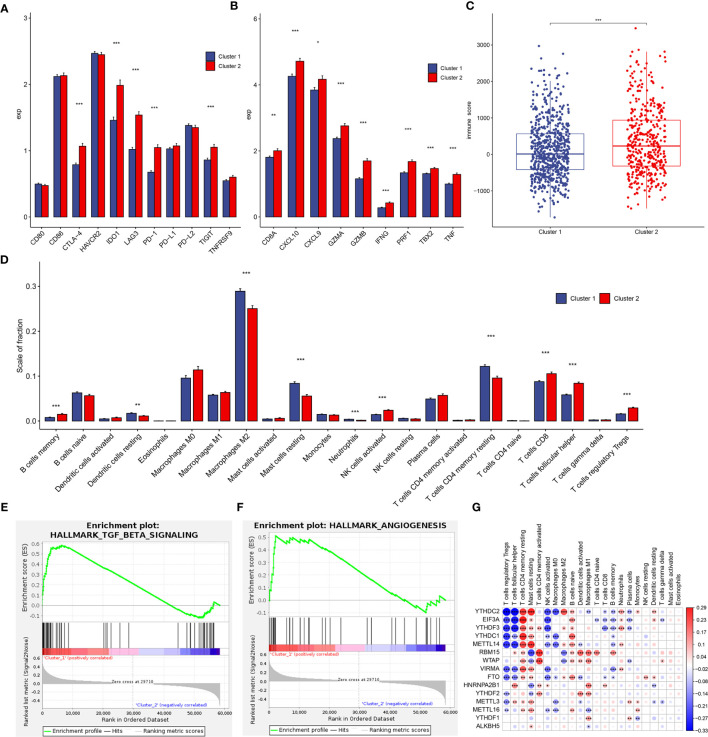
Tumor immune microenvironment (TIME) cell infiltration characteristics and immune-related gene expression in two clusters. **(A)** Differential expression of immune checkpoint-related genes in two clusters. **(B)** Differential expression of immune activation-related genes in two subtypes. **(C)** Immunoscore in the cluster 1 and cluster 2 subtypes. **(D)** The infiltrating levels of 22 immune cell types in cluster 1 and cluster 2 from the TCGA cohort. **(E, F)** GSEA results revealed that TGF-β signaling **(E)** and angiogenesis **(F)** were significantly enriched in cluster 1. **(G)** The correlation between each TIME infiltration cell type and each m^6^A regulator using spearman analyses. Negative correlation was marked with blue and positive correlation with red. *p < 0.05, **p < 0.01, ***p < 0.001.

To inquiry the involvement of immune regulation with m^6^A RNA methylation, we analyzed differential expression of several immune checkpoints, such as CD80, CD86, CTLA-4, HAVCR2, IDO1, LAG3, PD-1, PD-L1, PD-L2, TIGIT, and TNFRSF9 in two subtypes defined by m^6^A regulators (25). The results revealed CTLA-4, IDO1, LAG3, PD-1, TIGIT were significantly upregulated in cluster 2 compared to cluster 1 (**Figure 5A**). Interestingly, all immune activation transcripts CD8A, CXCL9, CXCL10, TNF, IFNG, TBX2, GZMB, PRF1, and GZMA also had higher expression in cluster 2 (**Figure 5B**) (25). This suggested that cluster 2 was significantly related to immune activation status, so had longer survival compared to cluster 1 (**Figure 4H**).

To investigate the effect of m^6^A regulators on the tumor immune microenvironment (TIME) of breast cancer, we first assessed the immunoscore between cluster 1 and cluster 2, and cluster 2 had higher immunoscore (**Figure 5C**). Subsequently, the immune infiltrate fraction of 22 immune cell types was analyzed. Cluster 1 showed higher infiltration levels of dendritic cells resting, macrophages M2, mast cells resting, neutrophils, and T cells CD4 memory resting, whereas cluster 2 was remarkably rich in B cells memory, NK cells activated, T cells CD8, T cells follicular helper, and T cells regulatory Tregs (**Figure 5D**). These results suggested a stronger immune activation of T cells and NK cells in cluster 2, consistently, and patients with this m^6^A modification pattern had longer survival (**Figure 4H**). The IHC results validated a part of 39 clinical breast tumor samples had distinct CD4^+^, CD8^+^, and regulatory T cell infiltration (**Supplementary Figure S5**). However, patients in cluster 1 similarly represented a degree of immune cell infiltration. We speculated that the profile of cluster 1 was the immune-excluded phenotype, which was also characterized by the presence of abundant immune cells, but the immune cells did not penetrate the parenchyma of these tumors but instead were retained in the stroma that surrounded nests of tumor cells (26). Therefore, reactive stroma in cluster 1 might be represented by increased influence of immunosuppression. The GSEA analyses revealed that stromal activation-related pathways were significant enrichment in cluster 1, such as TGF-β signaling (p = 0.0019, **Figure 5E**) and angiogenesis (p = 0.0495, **Figure 5F**). In addition, the infiltration of inactivated innate immune cells of cluster 1 in our results was in accordance with the characterization of the “innate immune-inactivated” cluster described by Xiao et al. (27). Therefore, these results verified our inference that the patients in cluster 1 had immune-excluded phenotype. Although patients of cluster 2 had a survival advantage, the profile of this cluster was more like the immune-inflamed phenotype, which was characterized by the presence in the tumor parenchyma of both CD4- and CD8-expressing T cells, often accompanied by myeloid cells and monocytic cells, and the immune cells were positioned in proximity to the tumor cells (26). High PD-1, IDO1, and TNF expression and high innate and adaptive immune cells infiltration in cluster 2 accorded with the phenotype feature of inflamed tumors (**Figures 5A, B**) (26, 27).

We then explored the correlations between each immune infiltration cell type and each m^6^A regulator using spearman’s correlation analyses. The results showed that YTHDC2 was correlated with 15 infiltrating immune cells (p < 0.05), followed by EIF3A and YTHDF3 (**Figure 5G**). This indicated potential functions of m^6^A readers in regulating intratumoral antitumor immune response *via* m^6^A methylation.

### m^6^A Phenotype-Related Gene Signatures and Clinical Correlation in Breast Cancer

**Figure 6:**
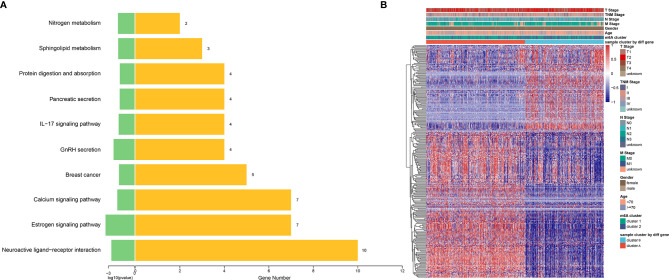
Construction of differential expression of m^6^A gene signatures and functional annotation. **(A)** Functional annotation for 533 m^6^A-related genes using KEGG pathway analysis. The yellow barplots represented the number of genes enriched; the green barplots represented p-value. **(B)** Unsupervised clustering of overlapping m^6^A phenotype-related DEGs to classify breast cancer patients into different subtypes, termed as cluster A and B, respectively. The gene signature subtypes, m^6^A clusters, tumor stage, gender, and age were used as patient annotations.

Although the consensus clustering based on 15 m^6^A regulators’ expression classified breast cancer patients into two m^6^A modification clusters, the underlying m^6^A phenotype-related transcriptional expression differences within these two clusters were not well known. We found 533 differentially expressed genes among the two m^6^A modification patterns, including 273 upregulated and 260 downregulated genes. There were 211 oncogenes high-expressed in cluster 1, including the well-known KRAS, NRAS, BCL2, EGFR, ABL1, MET, KIT, MDM2, ETS1, PIK3CA, and cluster2 high-expressed 175 oncogenes, including HRAS, JUN, AKT1 (**Supplementary Table S8**). These results indicated that more oncogenes were highly expressed in cluster 1, which had a worse prognosis (**Figure 4H**). KEGG pathway analysis of these differential genes revealed that enrichment of pathways remarkably related to estrogen signaling, IL-17 signaling, prolactin signaling pathways, and breast cancer, which confirmed that m^6^A modification played important roles in breast-related hormone signaling and immune regulation (**Figure 6A**). To further validate this regulation mechanism, we then performed unsupervised consensus clustering based on the further filtered m^6^A phenotype-related genes in order to classify these patients into different transcriptomic subtypes. Consistent with the clustering of m^6^A modification patterns, the unsupervised clustering also revealed two distinct m^6^A gene signature subtypes, and we named m^6^A gene cluster A-B, respectively (**Figure 6B**). Therefore, the m^6^A phenotype-related gene signatures could be used for subtyping in breast cancer.

## Discussion

To date more than 170 types of RNA modification have been identified in various RNA types, including those in mRNA, tRNA, rRNA, and other non-coding RNAs (28). Among them, m^6^A is the most common internal RNA modification in mRNA and has been found to be highly conserved in mammals and other eukaryotic species (28). Although first discovered in the 1970s (29), the absence of detection methods and the ambiguity of molecular mechanisms made the progress of this field slow. The identification of numerous m^6^A RNA methylation regulators including “writers” (methyltransferases), “erasers” (demethylases), and “readers” (recognition proteins) unveiled its functional importance of this epitranscriptomic modification in various cell types. At the molecular level, the m^6^A modification functions at almost lifetime of the mRNA metabolism, including alternative splicing, export, and translation, and regulates mRNA decay (30). These m^6^A regulators participate in tumor cell differentiation, angiogenesis, immune response, inflammatory response, or carcinogenesis *via* regulating expression of tumor-related genes dependent on its m^6^A modification (22). However, the roles of m^6^A regulators have been only sporadically reported in breast cancer. Several previously published reports revealed that m^6^A methyltransferases and demethylases both had oncogenic functions by regulating different targets in breast cancer, including METTL3 (16), METTL14 (17), FTO (15), and ALKBH5 (31). Therefore, simultaneously systematic study of biological value of most of these regulators is necessary in breast cancer.

Considering the role of genetic alternations in tumorigenesis, we firstly focused on the possibility of genomic variations of 15 chosen m^6^A regulators in breast cancer based on TCGA dataset. To our knowledge, this was the first time to study mutations of m^6^A RNA methylation regulators in breast cancer. Although few samples (6.39%) of breast cancer showed somatic mutations in these m^6^A regulators, most samples had CNV amplification events with relatively high frequency. In gastric cancer, 101 of 433 samples (23.33%) experienced mutations of 21 m^6^A regulators; however, most regulators had CNV amplification with relatively lower frequency than breast cancer (25). In head and neck squamous cell carcinoma (HNSCC), only 41 (8.1%) of 506 samples had mutation events in any of the 10 m^6^A regulatory genes, and the levels of CNV events ranged from 23.58 to 57.36% (32), which were lower than that of most regulators in our data. These results suggested tumor heterogeneity in various cancers. Interestingly, the reader gene YTHDF3 showed higher frequency of CNV events in all three cancers. However, only METTL3 deep or shallow deletion showed poorer overall survival in all regulators’ CNV events. Therefore, the CNV data based on exome sequencing from TCGA database might be further validated in more clinical samples by other CNV detection methods to rule out false positive results.

In this study, the expression of m^6^A “writers” METTL3, METTL14, and “erasers” FTO were downregulated in 112 tumor samples compared to the paired normal controls based on TCGA breast cancer dataset. It seemed that these m^6^A regulators did not function as oncogenes with high expression reported by other studies (15–17). The survival analysis based on the expression levels of 15 m^6^A regulators found that upregulated m^6^A readers YTHDF1 and YTHDF3 predicted poor survival. Coincidentally, Anita et al. recently showed that YTHDF1 and YTHDF3 aberrations were associated with metastasis and predicted poor prognosis in breast cancer patients (33). Chang et al. reported YTHDF3 could promote breast cancer brain metastasis by inducing the translation of m^6^A-enriched gene transcripts (34). However, the potential roles of YTHDF1 and YTHDF3 revealed in this study were merely validated in limited data and clinical samples. The molecular mechanism that how these two m^6^A readers functioned in breast cancer needs to be studied in the future.

Two clusters of patients with breast cancer were uncovered based on the expression levels of 15 m^6^A regulators, and most of these regulators revealed significant difference in two clusters. Although molecular heterogeneity and different clinicopathological features existed in most patients, the overall survival of patients in these two clusters revealed significant difference. Previous studies though identified breast cancer subtypes based on genomic, transcriptomic, or metabolic profiling (35–37). The cluster strategy by epitranscriptomic data based on the expression levels of m^6^A regulators also provided novel idea to improve the power of diagnosis, prognosis, and precision-focused, personalized treatment for breast cancer. Similarly, the molecular information of m^6^A regulators were applied to the subtyping of distinct cancers, including gastric cancer (25), colon cancer (38), and clear-cell renal carcinoma (39).

Recently, immunotherapy is emerging as a new treatment modality in breast cancer. Immune checkpoint inhibitor therapy by targeting the PD-1 axis has provided promising approaches in the field of breast cancer treatment (40, 41). The m^6^A RNA methylation was newly found to function in controlling various aspects of immunity, including immune recognition, activation of innate and adaptive immune responses, especially in antitumor immune responses (42). Therefore, we hypothesized that breast cancer patients with different m^6^A patterns might have different immune responses. Consensus clustering of 15 m^6^A regulators could divide 1,079 breast cancer patients into two subsets, and most m^6^A regulators had significantly differential expression in two clusters. Surprisingly, a lot of immune checkpoint genes (PD-1, CTLA-4) and immune activation transcripts (CD8A, IFNG) were high-expressed in cluster 2 compared to cluster 1. Immune activation status in cluster 2 implied better antitumor responses. Our results also revealed the overall survival of cluster 2 was longer than that of cluster 1. Furthermore, the significant survival differences between the two clusters might be related to the more important role of the higher immunoscore in cluster 2.

The tumor microenvironment is the primary location in which tumor cells and the host immune system interact. Tumor microenvironment plays an essential regulatory role in tumorigenesis and development, and its heterogeneity can influence patient prognosis and therapeutic response. Different lymphocytes infiltrate into the tumor microenvironment, and they can modulate tumor immune responses in both primary tumors and metastatic sites (43, 44). In the present study, the differences of immune cell infiltration in two clusters demonstrated that the m^6^A modification patterns could shape different TIME landscapes. Therefore, a comprehensive analysis of the m^6^A modification patterns will enhance our understanding of TIME cell infiltrating features. We speculated that cluster 1 and cluster 2 exhibited immune-excluded and immune-inflamed phenotypes, respectively. In the immune-excluded phenotype, the stroma may be limited to the tumor capsule or might penetrate the tumor itself, making it seem that the immune cells are actually inside the tumor (26). The GSEA results verified stromal activation signaling pathways were enriched in cluster 1. The profile of immune-inflamed phenotype suggests the presence of a pre-existing antitumor immune response that was arrested, and inflamed tumors also contain proinflammatory cytokines that should provide a more favorable environment for T-cell activation and expansion, including type I and type II IFNs, tumor-necrosis factor (TNF)-α (26). Our results also suggested cluster 2 had higher expression of some proinflammatory cytokines. Furthermore, the TIME phenotypes of these two clusters identified by m^6^A RNA methylation regulators in our study were highly consistent with that of two clusters (cluster 2 and 3) in a triple-negative breast cancer study (27). Cluster 3 (immune-inflamed cluster) had significantly better relapse-free survival and overall survival than cluster 2 (innate immune-inactivated cluster), which also confirmed the prognostic analysis in our study (27). The different immune phenotypes revealed by these two clusters in current analysis need to be explored further in clinical samples.

In conclusion, this study systematically evaluated the genetic variations and gene expression levels of 15 m^6^A regulators in breast cancer. The CNV alternations had important effects on gene expression of m^6^A RNA methylation regulators. Several m^6^A regulators had significantly differential mRNA and protein expression in breast tumor and adjacent tissues, and m^6^A readers YTHDF1 and YTHDF3 might be good survival predictors. Two breast cancer clusters (cluster 1 and cluster 2) were identified *via* the consensus clustering for m^6^A regulators. These clusters represented different survival situations, which could be further explained by the diversity of tumor immune microenvironment in these two subgroups. The clusters of breast cancer defined by m^6^A regulator patterns also showed different cancer-immune phenotypes. Therefore, identifying m^6^A regulator pattern might be helpful to uncover the mechanism underlying tumor microenvironment and immune responses. Our findings provided novel insights for improving breast cancer patients’ clinical response to immunotherapy in the future.

## Data Availability Statement

The datasets presented in this study can be found in online repositories. The names of the repository/repositories and accession number(s) can be found in the article/**Supplementary Material**.

## Ethics Statement

The studies involving human participants were reviewed and approved by the Ethics Committee of Qilu Hospital (Qingdao). The patients/participants provided their written informed consent to participate in this study.

## Author Contributions

GZ designed the paper and performed bioinformatics analysis. JA completed the data collection from the database. QP and WG completed the data verification and partial information analysis. HS and QZ completed the immunohistochemical experiment. HG critically reviewed and edited the manuscript. All authors contributed to the article and approved the submitted version.

## Funding

This work was supported by the Chinese National Natural Science Foundation Projects Grant No. 81572587 and Key projects of Qingdao science and technology plan Grant No. 19-6-1-4-nsh.

## Conflict of Interest

The authors declare that the research was conducted in the absence of any commercial or financial relationships that could be construed as a potential conflict of interest.

## Publisher’s Note

All claims expressed in this article are solely those of the authors and do not necessarily represent those of their affiliated organizations, or those of the publisher, the editors and the reviewers. Any product that may be evaluated in this article, or claim that may be made by its manufacturer, is not guaranteed or endorsed by the publisher.
