# Supplementary material for: Gene Signatures and Cancer-Immune Phenotypes Based on m6A Regulators in Breast Cancer
Source: Front Oncol. 2021 Nov 4;11:756412. doi: 10.3389/fonc.2021.756412 (PMC8600443; doi:10.3389/fonc.2021.756412)
Supplement: Supplementary file 2 [file DataSheet_2.pdf]

Supplementary file 2: High YTHDF3 expression in breast cancer tissues.

A

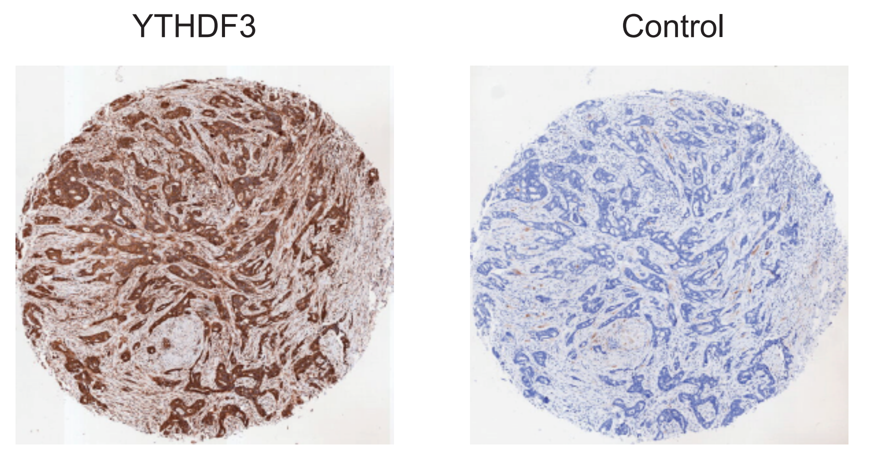

Representative images of immunostained with YTHDF3 or secondary antibody alone (right). (reference: PMID 26565812).

B

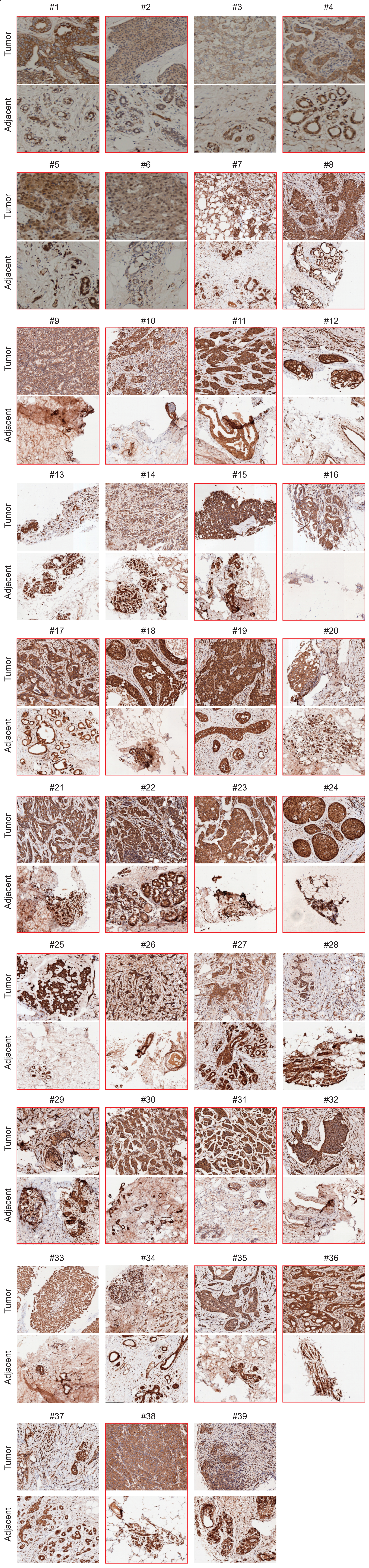

Immunohistochemical results of YTHDF3 in 39 pairs of samples, of which 30 pairs (red frame) showed a tendency of high expression in cancer tissues (assessed by positive optical density area and mean positive optical density by Image J software).
