# Supplementary material for: Gene Signatures and Cancer-Immune Phenotypes Based on m6A Regulators in Breast Cancer
Source: Front Oncol. 2021 Nov 4;11:756412. doi: 10.3389/fonc.2021.756412 (PMC8600443; doi:10.3389/fonc.2021.756412)
Supplement: Supplementary file 3 [file DataSheet_3.pdf]

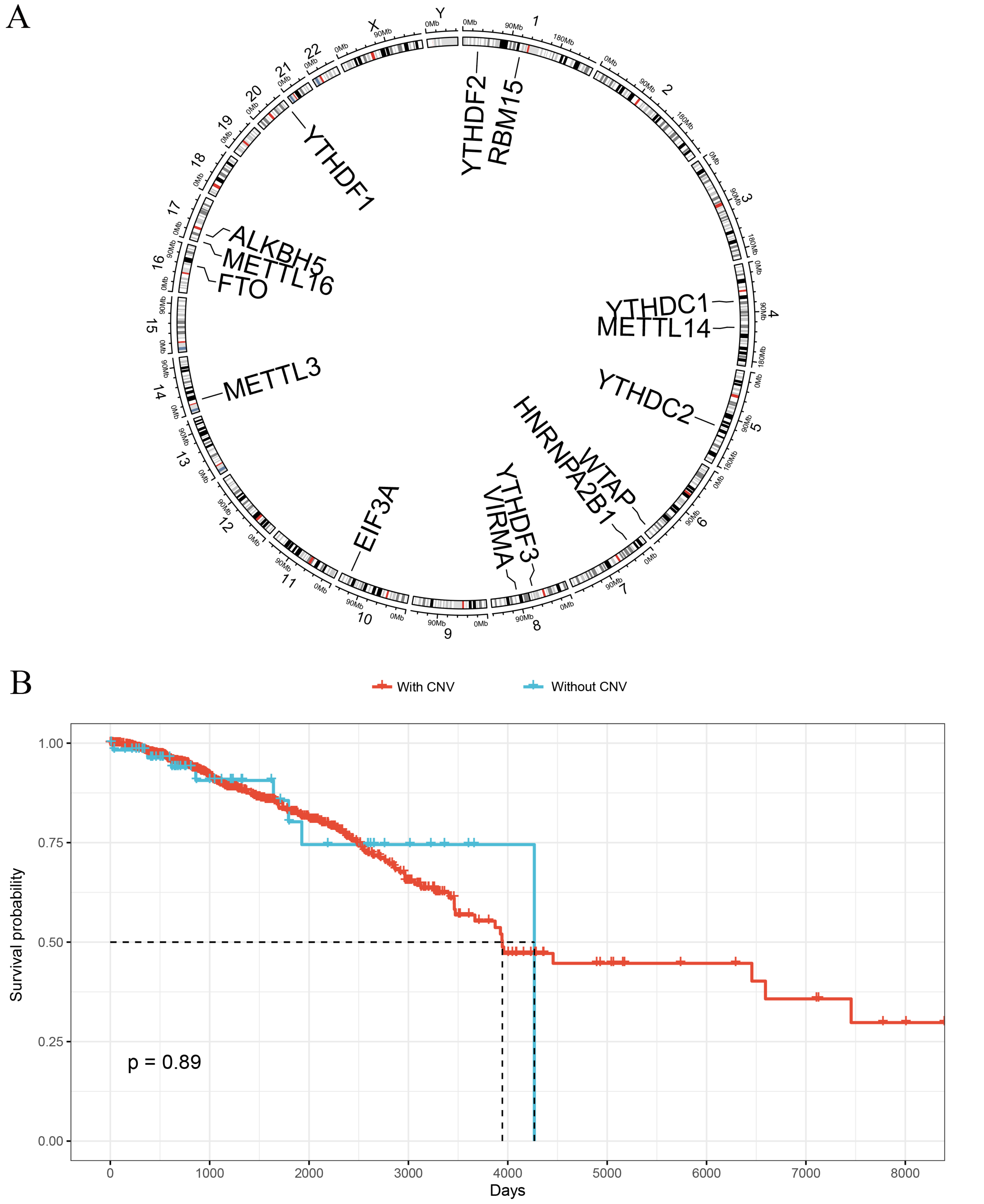

Figure S1 (A) The location of CNVs of 15 m6A regulators on chromosomes. (B) Overall survival of breast cancer patients with any CNVs of 15 m6A regulators or without CNVs.

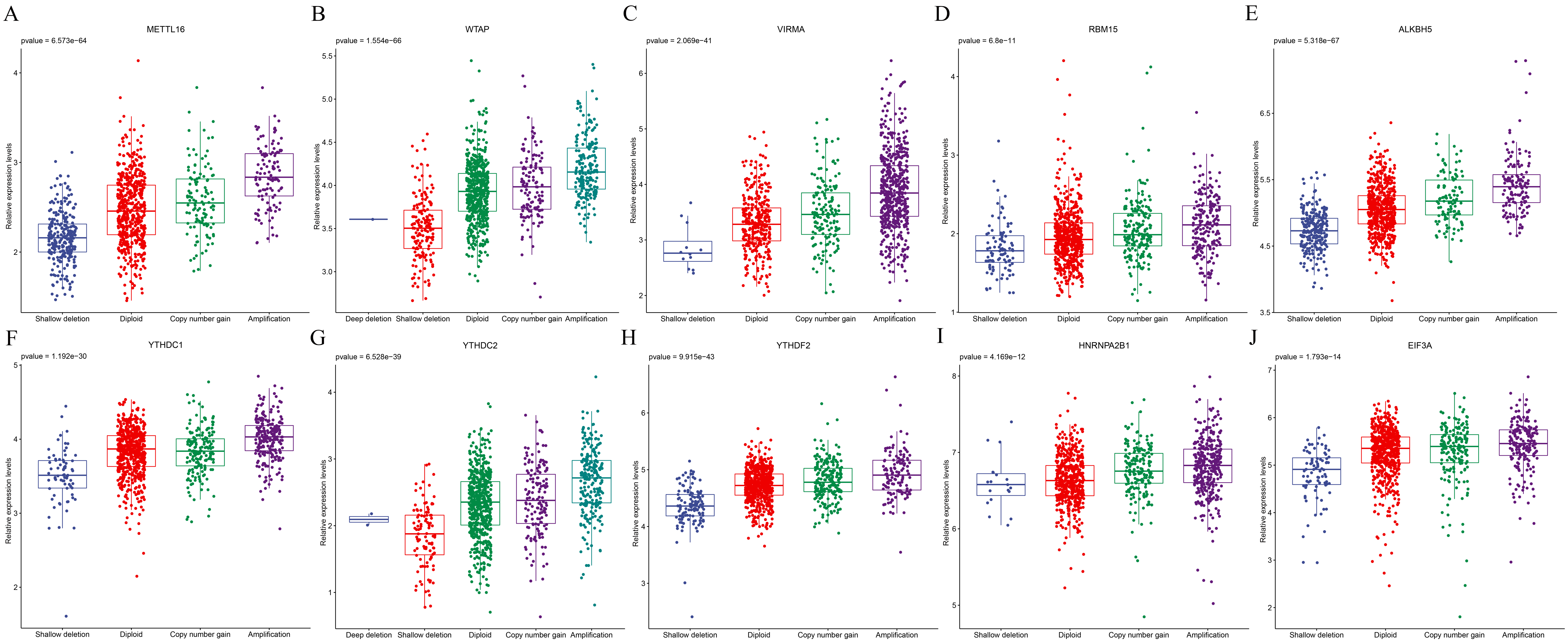

Figure S2 The correlations between different CNV patterns and mRNA expression levels of other 11 m6A regulators.

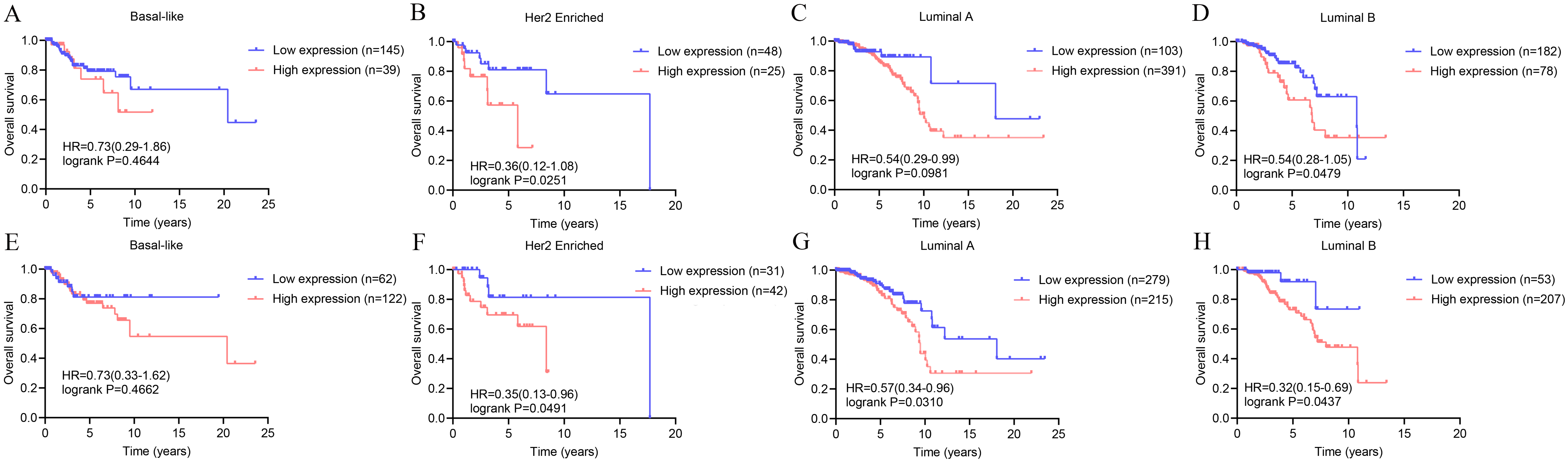

Figure S3 The correlation of YTHDF1 (A-D) or YTHDF3 (E-H) expression and overall survival of breast cancer patients with different molecular subtypes based on the HPA data.

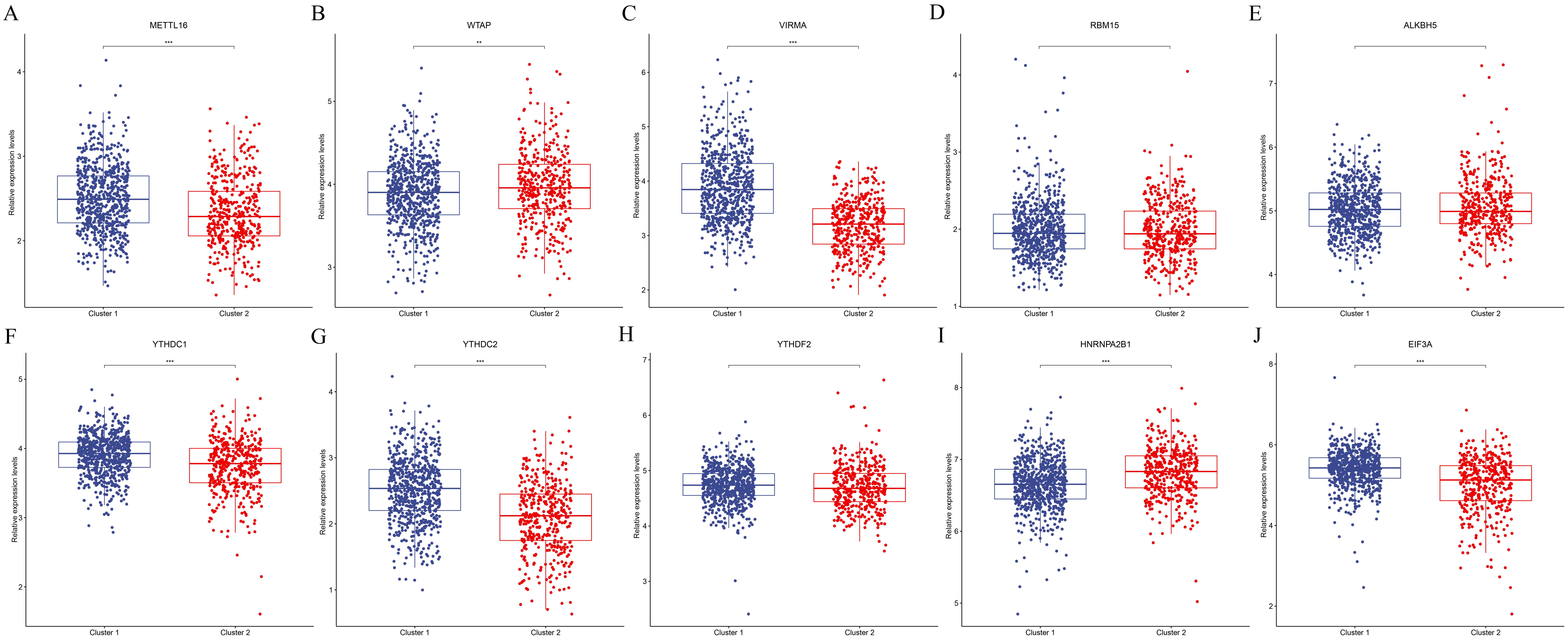

Figure S4 The relative expression levels of other 11 m6A regulators between cluster 1 and cluster 2.

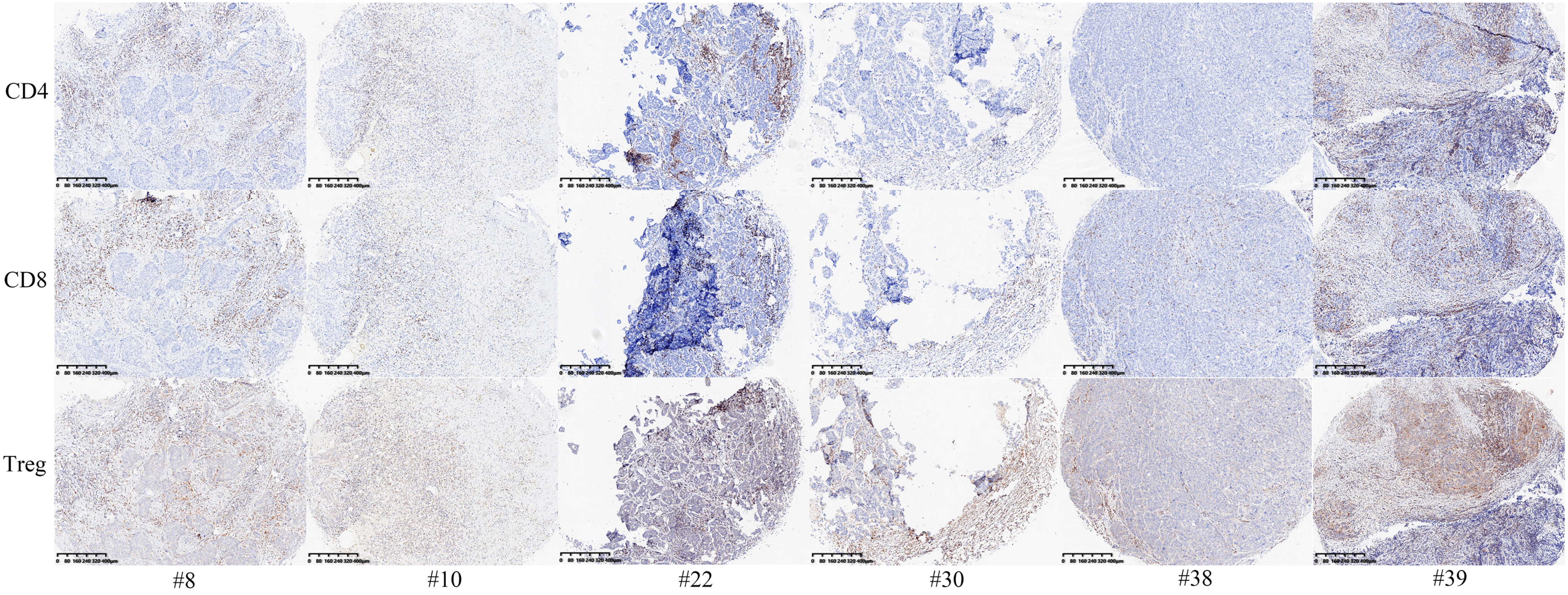

Figure S5 The IHC results of $CD4^{+}$, $CD8^{+}$ and regulatory T cell infiltration in 6 representative tumor samples of our clinical cohort.

Table S1 Clinical pathological parameters of breast cancer patients with or without CNV of m^6A regulators

		With CNV (%)	Without CNV (%)	p-value
No. of patients		997 (94.15)	62 (5.85)	
Age		58.4 ± 13.16	59.73 ± 13.75	0.46364152
Gender	female	986 (98.9)	62 (100)	0.405745616
	male	11 (1.1)	0 (0)	
Pathological stage	I	159 (16.13)	16 (25.81)	0.206232559
	II	571 (57.91)	31 (50)	
	III	225 (22.82)	15 (24.19)	
	IV	20 (2.03)	0 (0)	
	X	11 (1.12)	0 (0)	
T stage	T1	249 (24.97)	22 (35.48)	0.176761421
	T2	584 (58.58)	27 (43.55)	
	T3	124 (12.44)	11 (17.74)	
	T4	37 (3.71)	2 (3.23)	
	TX	3 (0.3)	0 (0)	
N stage	N0	466 (46.74)	34 (54.84)	0.119150738
	N1	330 (33.1)	19 (30.65)	
	N2	114 (11.43)	2 (3.23)	
	N3	68 (6.82)	7 (11.29)	
	NX	19 (1.91)	0 (0)	
M stage	M0	829 (83.15)	53 (85.48)	0.49486313
	M1	22 (2.21)	0 (0)	
	MX	146 (14.64)	9 (14.52)	

Table S2 Clinical pathological parameters of breast cancer patients with or without CNV of VIRMA

		With CNV (%)	Without CNV (%)	p-value
No. of patients		720 (67.99)	339 (32.01)	
Age		58.47 ± 13.16	58.5 ± 13.29	0.974691861
Gender	female	711 (98.75)	337 (99.41)	0.322992772
	male	9 (1.25)	2 (0.59)	
Pathological stage	I	103 (14.45)	72 (21.49)	0.051931258
	II	422 (59.19)	180 (53.73)	
	III	164 (23)	76 (22.69)	
	IV	16 (2.24)	4 (1.19)	
	X	8 (1.12)	3 (0.9)	
T stage	T1	166 (23.06)	105 (30.97)	0.032221406
	T2	436 (60.56)	175 (51.62)	
	T3	89 (12.36)	46 (13.57)	
	T4	26 (3.61)	13 (3.83)	
	TX	3 (0.42)	0 (0)	
N stage	N0	341 (47.36)	159 (46.9)	0.22622592
	N1	230 (31.94)	119 (35.1)	
	N2	83 (11.53)	33 (9.73)	
	N3	49 (6.81)	26 (7.67)	
	NX	17 (2.36)	2 (0.59)	
M stage	M0	608 (84.44)	274 (80.83)	0.109206379
	M1	17 (2.36)	5 (1.47)	
	MX	95 (13.19)	60 (17.7)	

Table S3 Clinical pathological parameters of breast cancer patients with or without CNV of YTHDF3

		With CNV (%)	Without CNV (%)	p-value
No. of patients		665 (62.80)	394 (37.20)	
Age		58.78 ± 13.18	57.96 ± 13.22	0.328699076
Gender	female	658 (98.95)	390 (98.98)	0.953726268
	male	7 (1.05)	4 (1.02)	
Pathological stage	I	97 (14.72)	78 (20.05)	0.121763721
	II	383 (58.12)	219 (56.3)	
	III	156 (23.67)	84 (21.59)	
	IV	16 (2.43)	4 (1.03)	
	X	7 (1.06)	4 (1.03)	
T stage	T1	151 (22.71)	120 (30.46)	0.03022639
	T2	403 (60.6)	208 (52.79)	
	T3	82 (12.33)	53 (13.45)	
	T4	26 (3.91)	13 (3.3)	
	TX	3 (0.45)	0 (0)	
N stage	N0	314 (47.22)	186 (47.21)	0.276135578
	N1	208 (31.28)	141 (35.79)	
	N2	78 (11.73)	38 (9.64)	
	N3	50 (7.52)	25 (6.35)	
	NX	15 (2.26)	4 (1.02)	
M stage	M0	558 (83.91)	324 (82.23)	0.346500944
	M1	16 (2.41)	6 (1.52)	
	MX	91 (13.68)	64 (16.24)	

Table S4 Clinical pathological parameters of breast cancer patients with or without CNV of YTHDF1

		With CNV (%)	Without CNV (%)	p-value
No. of patients		633 (59.77)	426 (40.23)	
Age		58.01 ± 13.19	59.18 ± 13.19	0.15486193
Gender	female	624 (98.58)	424 (99.53)	0.133911485
	male	9 (1.42)	2 (0.47)	
Pathological stage	I	85 (13.58)	90 (21.33)	0.004131622
	II	373 (59.58)	229 (54.27)	
	III	149 (23.8)	91 (21.56)	
	IV	15 (2.4)	5 (1.18)	
	X	4 (0.64)	7 (1.66)	
T stage	T1	135 (21.33)	136 (31.92)	0.003939658
	T2	387 (61.14)	224 (52.58)	
	T3	83 (13.11)	52 (12.21)	
	T4	26 (4.11)	13 (3.05)	
	TX	2 (0.32)	1 (0.23)	
N stage	N0	293 (46.29)	207 (48.59)	0.248776766
	N1	211 (33.33)	138 (32.39)	
	N2	79 (12.48)	37 (8.69)	
	N3	40 (6.32)	35 (8.22)	
	NX	10 (1.58)	9 (2.11)	
M stage	M0	531 (83.89)	351 (82.39)	0.108629081
	M1	17 (2.69)	5 (1.17)	
	MX	85 (13.43)	70 (16.43)	

Table S5 The p-value of the significant correlations of clinical pathological parameters and CNVs of other m^6A regulators

m^6A regulators	Pathological stage	T stage
EIF3A	0.005426965	
HNRNPA2B1	0.000723942	0.000318173
METTL3		0.014999734
METTL14	0.016572431	0.000096613
WTAP	0.028574962	
YTHDC1		0.017155734
YTHDC2	0.016516836	0.038678051

Table S6. Clinical data of 39 patients involved in this manuscript

Patients[1]	Gender	Age	Time of first diagnosis[2]	Pathology results[3]	Immunohistochemical results[4]	TNM[5]	Molecular subtyping[6]	Surgical method[7]	Chemotherapy regimens[8]	Survival state[9]
#1	Female	38	7 Sept 2018	BIDC	ER(++,70%),PR(++～+++,70%), Her2(+++),Ki67 (+,80%)	pT3N0M0 IIIB	Lumina B	MRM	EC*4-T*4+ Trastuzumab	Died of multiple metastases
#2	Female	38	24 Sept 2018	BIDC	ER(+++,90%),PR(+,30%), Her2(+～++),Ki67 (+,20%)	pT2N0M0 IIA	Lumina B	MRM	TC*4	Progression-free
#3	Female	45	20 Jul 2018	BIDC	ER(-),PR(-), Her2(++),Ki67 (+,50%)	ypT3N3M0 IIIC	Her2 overexpressing	RM	(EC+T) *6+Trastuzumab	Died of multiple metastases
#4	Female	54	24 Aug 2018	BIDC	ER(-),PR(-), Her2(+++),Ki67 (+,30%)	pTxN3M0 IIIC	Her2 overexpressing	MRM	EC*4-T*4+ Trastuzumab	Progression-free
#5	Female	47	12 Mar 2018	BIDC	ER(-),PR(-), Her2(-),Ki67 (+,80%)	pT2N0M0 IIA	TNBC	MRM	ET *6	Died of multiple metastases
#6	Female	53	25 Dec 2017	BLBC	ER(-),PR(-), Her2(-),Ki67 (30% ~ 40%)	pT2N1M0 IIB	TNBC	MRM	EC*4−T*4	Progression-free
#7	Female	59	6 Jun 2020	BICC	ER(++,20%),PR(-), Her2(+ ~ ++),Ki67(+,20%)	ypT3N0M0 IIIC	Lumina B	MRM	ET*6-NP*4	Progression-free
#8	Female	46	3 Jul 2020	BIC	ER(++,70%),PR(++ ~ +++,60%), Her2(+++),Ki67 (+,30%)	pT1N0M0 IA	Lumina B	MRM	EC*4−T*4+ Trastuzumab	Progression-free
#9	Female	56	6 Jul 2020	BIDC	ER(+++,20%),PR(++,90%), Her2(-),Ki67 (+,10%)	pT2N0M0 IIA	Lumina A	MRM	Unknown	Progression-free
#10	Female	55	10 Jul 2020	BMC	ER(-),PR(-), Her2(++),Ki67 (+,50%)	pT1N0M0 IA	Her2 overexpressing	MRM	EC*4−T*4+ Trastuzumab	Progression-free
#11	Female	82	14 Jul 2020	BEPC	ER(++ ~ +++,90%),PR(++ ~ +++,90%), Her2(-),Ki67 (+,15%)	pT2N0M0 IIA	Lumina B	MRM	Unknown	Progression-free
#12	Female	36	19 Jul 2020	BILC	ER(+++,95%),PR(++ ~ +++,90%), Her2(+ ~ ++),Ki67 (+,20%)	ypT2N1M0 IIB	Lumina B	MRM	ET*6	Progression-free

#13	Female	55	23 Jul 2020	BIC	ER(+++,80%),PR(++,40%), Her2(-),Ki67 (+,50%)	pT1N1M0 IIA	Lumina B	MRM	Unknown	Progression-free
#14	Female	63	24 Jul 2020	BILC	ER(+++,90%),PR(+++,70%), Her2(+),Ki67 (+,20%)	pT2N1M0 IIB	Lumina B	MRM	CT*6	Progression-free
#15	Female	52	27 Jul 2020	BIDC	ER(++ ~ +++,80%), PR(++ ~ +++,70%), Her2(-),Ki67 (+,10%)	pT1N0M0 I A	Lumina A	MRM	Unknown	Progression-free
#16	Female	69	4 Aug 2020	BIDC	ER(++ ~ +++,90%),PR(-), Her2(+ ~ ++),Ki67 (+,10%)	pT1N0M0 I A	Lumina B	MRM	Unknown	Progression-free
#17	Female	46	5 Aug 2020	DCIS	ER(+++,90%),PR(++ ~ +++,90%), Her2(+),Ki67 (+,20%)	pTisN0M0 0	Lumina B	MRM	Unknown	Progression-free
#18	Female	40	8 Aug 2020	BIC	ER(+++,70%),PR(+++,90%), Her2(++),Ki67 (+,5%)	pT1N1M0 IIA	Lumina B	MRM	EC*6	Progression-free
#19	Female	84	22 Aug 2020	BIDC	ER(+++,90%),PR(-), Her2(-),Ki67 (+,15%)	pT1N0M0 I A	Lumina B	MRM	Unknown	Progression-free
#20	Female	56	23 Aug 2020	DCIS	ER(-),PR(-), Her2(+++),Ki67 (+,20%)	pTisN0M0 0	Her2 overexpressing	MRM	Unknown	Progression-free
#21	Female	29	29 Aug 2020	BIC	ER(-),PR(++,5%), Her2(-),Ki67 (+,50%)	pT1N0M0 I A	Lumina B	BCT	Unknown	Progression-free
#22	Female	48	5 Sept 2020	BIC	ER(++ ~ +++,80%), PR(++ ~ +++,80%), Her2(-),Ki67 (+,60%)	pT2N3M0 IIIB	Lumina B	MRM	Unknown	Progression-free
#23	Female	63	9 Sept 2020	BLBC	ER(-),PR(-), Her2(-),Ki67 (+,30%)	pT2N1M0 IIB	TNBC	MRM	Unknown	Progression-free
#24	Female	57	10 Sept 2020	BIC	ER(+++,90%),PR(+ ~ ++,30%), Her2(-),Ki67 (+,10%)	pT2N0M0 IIA	Lumina A	MRM	TC*4	Progression-free
#25	Female	62	12 Sept 2020	IMPC	ER(++ ~ +++,90%), PR(++,15%), Her2(++),Ki67 (+,20%)	ypT2N2M0 IIIA	Lumina B	RM	ET*6	Progression-free
#26	Female	57	14 Sept 2020	IMPC	ER(+++,90%),PR(+++,90%),	pT1N1M0	Lumina B	MRM	Unknown	Progression-free

					Her2(+),Ki67 (+,20%)	IIA				
#27	Female	73	25 Sept 2020	BMA	ER(+++,80%),PR(+ ~ ++,30%), Her2(++),Ki67 (+,10%)	pT2N0M0 IIA	Lumina B	MRM	Unknown	Progression-free
#28	Female	45	14 Oct 2020	BLBC	ER(-),PR(-), Her2(-),Ki67 (+,80%)	pT1N1M0 IIA	TNBC	MRM	EC*4-T*4	Progression-free
#29	Female	62	17 Oct 2020	BIC	ER(++ ~ +++,90%), PR(-), Her2(+++),Ki67 (+,20%)	pT1N0M0 I A	Lumina B	MRM	Trastuzumab	Progression-free
#30	Female	46	22 Oct 2020	BIC	ER(++ ~ +++,90%),PR(++ ~ +++,90%), Her2(+),Ki67 (+,20%)	pT1N0M0 I A	Lumina B	MRM	No chemotherapy	Progression-free
#31	Female	51	27 Oct 2020	IMPC	ER(++ ~ +++,80%),PR(+++,80%), Her2(+ ~ ++),Ki67 (+,10%)	pT1N0M0 I A	Lumina B	MRM	TC*4	Progression-free
#32	Female	62	28 Oct 2020	DCIS	ER(+++,90%),PR(+ ~ ++,40%), Her2(+),Ki67 (30%)	pT1N0M0 IA	Lumina B	MRM	Unknown	Progression-free
#33	Female	59	13 Nov 2020	BIDC	ER(++ ~ +++,90%), PR(++ ~ +++,70%), Her2(+),Ki67 (+,40%)	pT1N2M0 IIIA	Lumina B	MRM	EC*4-T*4	Progression-free
#34	Female	57	15 Nov 2020	BILC	ER(+++,90%),PR(++,50%), Her2(+),Ki67 (+,10%)	pT1N0M0 IA	Lumina B	MRM	Unknown	Progression-free
#35	Female	36	20 Nov 2020	BIC	ER(+ ~ ++,90%), PR(+ ~ ++,20%), Her2(+),Ki67 (+,20%)	pT3N3M0 IIIC	Lumina B	MRM	Unknown	Progression-free
#36	Female	53	22 Nov 2020	BIC	ER(++,90%),PR(++ ~ +++,90%), Her2(++),Ki67 (20%)	pT1N3M0 IIIC	Lumina B	MRM	EC*4-T*4	Progression-free
#37	Female	60	4 Dec 2020	BIC	ER(++ ~ +++,80%), PR(++,10%), Her2(++),Ki67 (+,30%)	pT1N0M0 IA	Lumina B	MRM	TCbHP	Progression-free
#38	Female	66	5 Dec 2020	BIDC	ER(+++,90%),PR(++ ~ +++,70%), Her2(+),Ki67 (+,20%)	pT1N2M0 IIIB	Lumina B	MRM	EC*4-T*4	Progression-free
#39	Female	44	9 Dec 2020	DCIS	ER(-),PR(-), Her2(++),Ki67 (10%)	pTisN0M0 0	Her2 overexpressing	MRM	Unknown	Progression-free

1 Data were collected from the Case Custodian Department of Qilu Hospital of Shandong University in Qingdao, Tissue samples from #1 to #6 were paraffin-embedded and the remaining tissue samples were fresh and frozen.

Correspondence address: 758 Hefei road, Qingdao, Shandong 200035 P.R. China.

Email: qlyyqdxcb@163.com.

2 The patient was first diagnosed with breast cancer at Qilu Hospital of Shandong University (Qingdao).

3 Invasive micropapillary carcinoma, IMPC; Basal-like breast carcinoma, BLBC; Breast invasive ductal carcinoma, BIDC; Breast mucinous adenocarcinoma, BMA; Breast invasive cribriform carcinoma, BICC; Breast invasive carcinoma, BIC; Breast metaplastic carcinoma, BMC; Breast encapsulated papillary carcinoma, BEPC; Breast invasive lobular carcinoma, BILC; Ductal carcinoma in situ, DCIS.

4 Data were obtained from the Department of Pathology, Qilu Hospital, Shandong University (Qingdao).

5 Staging is based on the AJCC Staging Manual, Edition 8. p stands for pathological stage, y is the stage after neoadjuvant chemotherapy.

6 Molecular typing was based on the typing scheme proposed by Perou et al.

Perou CM, Sørlie T, Eisen MB, et al. Molecular portraits of human breast tumours. Nature. 2000; 406(6797):747-752. doi:10.1038/35021093.

7 Radical mastectomy, RM; Modified radical mastectomy, MRM; Breast conserving therapy, BCT.

8 epirubicin, E; cyclophosphamide, C; paclitaxel liposome or nab-paclitaxel or docetaxel, T; docetaxel + carboplatin + trastuzumab + pertuzumab, TCbHP. The dose depends on the patient's weight and other indicators.

9 The follow-up time was up to March 30, 2021.

Table S7 Clinical pathological parameters of breast cancer patients in cluster 1 and cluster 2

		Cluster 1 (%)	Cluster 2 (%)	p-value
No. of patients		669 (62.00)	410 (38.00)	
Vital status	Alive	567 (84.75)	364 (88.78)	0.061985213
	Dead	102 (15.25)	46 (11.22)	
Gender	female	664 (99.25)	403 (98.29)	0.144446339
	male	5 (0.75)	7 (1.71)	
Pathological stage	I	121 (18.09)	60 (14.63)	0.103967087
	II	369 (55.16)	250 (60.98)	
	III	161 (24.07)	86 (20.98)	
	IV	9 (1.35)	11 (2.68)	
	X	9 (1.35)	3 (0.73)	
T stage	T1	191 (28.55)	85 (20.73)	0.079599664
	T2	372 (55.61)	254 (61.95)	
	T3	81 (12.11)	56 (13.66)	
	T4	23 (3.44)	14 (3.41)	
	TX	2 (0.3)	1 (0.24)	
N stage	N0	308 (46.04)	204 (49.76)	0.260058824
	N1	216 (32.29)	140 (34.15)	
	N2	82 (12.26)	35 (8.54)	
	N3	50 (7.47)	25 (6.1)	
	NX	13 (1.94)	6 (1.46)	
M stage	M0	577 (86.25)	324 (79.02)	0.006548281
	M1	9 (1.35)	11 (2.68)	
	MX	83 (12.41)	75 (18.29)	

Table S8 Distribution of oncogenes in two groups

Cluster 1			
ABL1	ENTPD5	MMS22L	SYK
ABL2	EPS8	MRAS	TAF1
ADAM9	ERBB3	MTDH	TAL2
AFF1	ERBB4	MTOR	TBC1D15
AFF3	ETS1	MYCL	TFCP2
AGFG1	ETV1	NCOA3	TNS4
AHCYL1	ETV3	NCOA4	TPD52
AKAP13	EVI5	NEDD4	TPR
AKAP9	FASN	NFIB	TRAF6
AR	FGF10	NRAS	TRIM32
ATF1	FGFR1	NSD1	TRIO
AXL	FNDC3B	NTRK3	UBE3C
BAALC	FOXO1	NUP214	URI1
BARD1	FUBP1	NUP98	USP22
BCKDHB	GALNT10	PBX1	USP4
BCL2	GLI3	PDGFRA	WAPL
BCL9L	GNA12	PDGFRB	WNT3
BIRC2	GNA13	PIK3CA	WNT5A
BMI1	GNAQ	PIK3R1	WWTR1
BRAF	GOLPH3	PLAG1	XIAP
CAMK1D	GOPC	PLAGL2	YAP1
CBL	GPM6B	PPM1D	YES1
CBLB	GREM1	PRDM10	YWHAG
CBLL1	HLF	PRKCA	YWHAQ
CCDC6	HSPA4	PRKCE	YWHAZ
CDC5L	IDH1	PRKCI	YY1
CDH1	IGF1R	PTCH1	ZFAND4
CDK14	IL7R	PTP4A1	ZMYM2
CDK6	INTS2	PTP4A2	ZNF217
CDK8	INTS7	PTPN11	ZNF268
CDON	INTS8	RAB11A	ZNHIT6
CEACAM6	JAK1	RAB18	EIF3E
CFLAR	JAK2	RAB22A	EIF4E
CHIC1	KAT6A	RAB23	EIF5A2
CHL1	KDM2A	RBM15	ELF4
CREB1	KDM4C	REL	ELK3
CRK	KDM5B	RERE	EML4
CRKL	KIT	RINT1	EMSY
CRLF2	KLF6	RIT1	MET
CSNK2A1	KLF8	ROCK1	MFHAS1
CSNK2A2	KMT2A	ROS1	MIR100HG
CT45A1	KRAS	RSF1	MIR99AHG
CTNNB1	LAPTM4B	RUNX1	MITF
CYP24A1	LMO3	RUNX1T1	MLANA
DCUN1D1	MALT1	SALL4	MLLT3
DDHD2	MAML2	SATB1	SMURF1
DDX6	MAP3K7	SEC62	SPAG9
DEK	MAPRE1	SERTAD2	SPARC
DIS3	MCC	SETBP1	SPRY2
DSG3	MDM2	SIRT1	STAT3
E2F5	MECOM	SKI	SUZ12
ECT2	MEF2C	SKIL	SWAP70
EGFR	MERTK	SKP2	

Cluster 2			
AGRN	GNAS	PRDM12	FOSL1
AKIRIN2	GPR132	PTMA	FOXM1
AKT1	GSK3A	PTP4A3	FRAT1
ARAF	H19	PTTG1	FUS
ARHGEF1	HAX1	PTTG2	FZD2
ARID3A	HDAC1	PVT1	GALR2
ASPSCR1	HES6	RAB8A	GNAI2
BANP	HMGA1	RAC1	PIM3
BAX	HMGN5	RALGDS	PLK1
BCL2A1	HNRNPA1	RARA	PLXNB1
BCL2L12	HOXD9	RASSF1	PML
BCL3	HRAS	RBM14	PPP1R13L
BIRC5	HSPA1A	RHOC	PPP1R14A
CAPG	HSPB1	RING1	PPP2R1A
CBX8	HULC	RPL23	ZBTB7A
CCDC28A	ID1	RRAS	ZFAS1
CCNB1	ID2	S100A4	
CCNB2	IKBKE	S100A8	
CCNE1	ILK	SBSN	
CCNL1	INPPL1	SERTAD1	
CDC25C	INTS1	SERTAD3	
CDK1	INTS3	SFPQ	
CDK4	JUN	SIRT6	
CDKN3	JUNB	SLC3A2	
CENPW	JUND	SNCG	
CKS1B	JUP	SOX2	
CMC4	KLF2	SPI1	
CRTC1	LAMTOR5	SQSTM1	
CTSZ	LMO2	SRSF2	
CTTN	MACROD1	SRSF3	
CYGB	MALAT1	SRSF6	
DAXX	MBD1	STMN1	
DDIT3	MCF2L	TAF15	
DNPH1	MCTS1	TALDO1	
DUSP12	MFNG	TAZ	
E2F1	MIEN1	TBC1D3C	
ECHS1	MIR17HG	TBC1D7	
EEF1D	MLLT1	TBX2	
EIF3I	MPST	TCF3	
ELAVL1	MST1R	TFE3	
ELL	NEAT1	TGFB1	
EPHA2	NFKB2	TLE1	
ERAS	NINL	TP73	
ESPL1	NME1	TRIM28	
ETV4	NUAK2	TRIM8	
EWSR1	PA2G4	TRIP6	
EZH2	PARK7	TXN	
FAM189B	PATZ1	TYMS	
FDPS	PBK	UBE2C	
FES	PBX2	UCHL1	
FGF8	PDGFB	UHRF1	
FGFR3	PELP1	WNT10B	
FGR	PIGU	YBX1	

Table S9 The primers of five m^{6}A regulators and GAPDH used in RT-qPCR

Gene Name	Forward Primer	Reverse Primer
METTL3	CAAGCTGCACTTCAGACGAA	GCTTGGCGTGTGGTCTTT
METTL14	AGAAACTTGCAGGGCTTCCT	TCTTCTTCATATGGCAAATTTTCTT
FTO	GGCTGCTTATTTCGGGACCT	GGACCGTAAAGAGCCTGGTG
YTHDF1	AGCACACAACCTCCATCTTCG	TTTCGACTCTGCCGTTCCTTG
YTHDF3	AGCCATGCGTAGGGAGAGAA	GGCATTTCCAGAGTCTACATCGT
GAPDH	GTCTCCTCTGACTTCAACAGCG	ACCACCCTGTTGCTGTAGCCAA

Table S10 Information about the antibodies used in this study

Name	Host	Dilution Ratio	URL
FTO	Rabbit	WB: 1:1000; IHC: 1:20	http://en.zen-bio.cn/prod_view.aspx?TypeId=127&Id=355908&FId=t3:127:3
METTL14	Rabbit	WB: 1:1000; IHC: 1:100	http://en.zen-bio.cn/prod_view.aspx?TypeId=126&Id=346821&FId=t3:126:3
METTL3	Rabbit	WB: 1:1000; IHC: 1:100	http://en.zen-bio.cn/prod_view.aspx?TypeId=126&Id=346854&FId=t3:126:3
YTHDF1	Rabbit	WB: 1:1000; IHC: 1:200	https://abclonal.com.cn/catalog/A18126
YTHDF3	Rabbit	WB: 1:1000; IHC: 1:200	https://abclonal.com.cn/catalog/A8395
FOXP3	Rabbit	IHC: 1:200	http://www.zen-bio.cn/prod_view.aspx?TypeId=136&Id=378388&FId=t3:136:3
CD4	Rabbit	IHC: working solution	http://www.zsbio.com/product/ZA-0519
CD8	Rabbit	IHC: working solution	http://www.zsbio.com/product/ZA-0508
VENTANA PD-L1(SP263)	Rabbit	IHC: working solution	https://diagnostics.roche.com/global/en/products/tests/ventana-pd-l1-_sp263-assay2.html
β-actin	Rabbit	WB: 1:1000	https://www.abcam.cn/beta-actin-antibody-ab8227.html
HRP	Goat Anti-Rabbit	IHC: 1:1000	https://www.abcam.cn/goat-rabbit-igg-hl-hrp-ab6721.html
